# Supplementary material for: Virulent properties and genomic diversity of Vibrio vulnificus isolated from environment, human, diseased fish
Source: Microbiol Spectr. 2024 Jun 11;12(7):e00079-24. doi: 10.1128/spectrum.00079-24 (PMC11218479; doi:10.1128/spectrum.00079-24)
Supplement: Table S1 — Primers for V. vulnificus genotypic characterization. [file spectrum.00079-24-s0001.docx]

**Table S1** Primers for *V. vulnificus* genotypic characterization

| **Primer** | **Gene target** | **Sequence (5' to 3')** | | **Ta (◦C)** | **Amplicon**  **size (bp)** | **Reference** |
| --- | --- | --- | --- | --- | --- | --- |
| **Man IIA F** | Mannitol fermentation operon | | GATGTTGGTGAACAACTTCTCTGC | 60.8 | 243 | (100) |
| **Man IIA R** |  |  | TCTGAAGCCTGTTGGATGCC |  |  |  |
| ***nanA*-F** | Sialic acid catabolism cluster | | TKATCGCCGCTCCYCATACA | 55 | 745 | (26) |
| ***nanA*-R** |  |  | GCAACGCCACCGTATTCAAC |  |  |  |
| **VV0316F** | YJ-like nab1 allele | | GGCCACCCCTTCAATTGAG | 60 | 435 | (79) |
| **VV0316R** |  |  | GTCGCATACACAACCGTGG |  |  |  |
| **VV0312F** | YJ-like nab2 allele | | CGACGAAGCACTGGCGTTTAA A | 61 | 986 |  |
| **VV0312R** |  |  | GCTCGAGCATCTCCCAATACT |  |  |  |
| **VV10803F** | CM-like nab1 allele | | TTATCGGCGACAAGGTGA | 57 | 346 |  |
| **VV10803R** |  |  | ATCCATTACATAGGCAAATATG |  |  |  |
| **VV10808F** | CM-like nab2 allele | | TATTCGTTTAGCCAAACAGTTGA | 57 | 902 |  |
| **VV10808R** |  |  | CCACTTCATCCCAACGCGTT |  |  |  |
| ***vcg* C-F** | Virulence-correlated gene clinical allele | | AGCTGCCGATAGCGATCT | 50 | 99 | (101) |
| ***vcg* C-R** |  |  | TGAGCTAACGCGAGTAGTGAG |  |  |  |
| ***vcg* E-F** | Virulence-correlated gene environmental allele | | CTCAATTGACAATGATCT | 55 | 278 |  |
| ***vcg* E-R** |  |  | CGCTTAGGATGATCGGTG |  |  |  |
| **VVA1612F** | Region XII, flanking region | | ACCCTGATCGTTGGCTACTC | 57 | 2,257 | (32) |
| **VVA1613R** | Region XII | | GGAGCGGTGTGATGGTGTTG |  |  |  |
| **VVA1625F** | Region XII | | CGGTCTGTGGTTTATCG | 47 | 1,822 |  |
| **VVA1625R** | Region XII | | TCGTTTCCAGTCGTCAC |  |  |  |
| **VVA1634F** | Region XII | | TGACACCCAACCTAGACCAC | 55 | 1,364 |  |
| **VVA1634R** | Region XII | | ATTGATGCCAACCTGAG |  |  |  |
| **VVA1636F** | Region XII | | TGTCCACGACTTGAACACG | 56 | 1,547 |  |
| **VVA1637R** | Region XII, flanking region | | AACATCAACCAGCGAGTCGAAC |  |  |  |
| **VVA1612bF** | Region XII, flanking region | | TGTGGAGAGCGGCAAGATCAAG | 61 | 1,200 |  |
| **VVA1637R** | Region XII, flanking region | | AACATCAACCAGCGAGTCGAAC |  |  |  |
