# Supplementary material for: Virulent properties and genomic diversity of Vibrio vulnificus isolated from environment, human, diseased fish
Source: Microbiol Spectr. 2024 Jun 11;12(7):e00079-24. doi: 10.1128/spectrum.00079-24 (PMC11218479; doi:10.1128/spectrum.00079-24)
Supplement: Table S2 — Primers for MLVA. [file spectrum.00079-24-s0002.docx]

**Table S2** Primers for MLVA analysis

| **Locus** | **Forward and Reverse primer (5’-3')** |  |
| --- | --- | --- |
| VV-0401 | F-FAM-AGACAGCACTGGTTCCTAATT  R- CTATCTGGAAATAAGCGAAGC |  |
| VV-1044 | F-HEX-GCTTTCACGCCAACCTTTCT  R-TCTGTCTCTGCTTGGTATCG |  |
| VV-1151 | F-NED-TCAATCGGGTCAGGAAGATAT  R- CGTGGTGAGATGAGCTTATAA |  |
| VV-2339 | F-FAM-CTTCATATCCATACTCCTGTG  R-GGTATCCTATCGTACTAGAAC |  |
| VV-2577 | F-HEX-ATATTGATCGTGCGGCATTAG  R- CCTTCGTATGTGAAGTTCGTT |  |
| VV-2785 | F-NED-GGTCTGAAGAGCAGTATTAAC  R- ATGCGTGAACATCAACCCTAT |  |
| VV1-1615 | F-ROX-CCGATAACATGATTTAGGCTC  R- TGTGATGGAGGTAACAAGTTG |  |
| VVA-0305 | F-FAM-ATTCGTACACAAATGTCTGCG  R- TCATCCAAATGCCTTCTTCTG |  |
| VVA-0375 | F-HEX-CGATTGGAAGACTTTTGTTGC  R- GTATGATAAAGCACTAGAGGC |  |
| VVA-0930 | F-NED-TGATTGGACAATATCGGATCG  R- GTGATGAACTGCGTATGGAAT |  |
| VVA-1475 | F-ROX-GACTACCAATACCAATATCGC  R- ACTAGGAAGTAGAGACTAGCT |  |
