# Supplementary material for: Virulent properties and genomic diversity of Vibrio vulnificus isolated from environment, human, diseased fish
Source: Microbiol Spectr. 2024 Jun 11;12(7):e00079-24. doi: 10.1128/spectrum.00079-24 (PMC11218479; doi:10.1128/spectrum.00079-24)
Supplement: Table S3 — Alleles detected across all 10 genes evaluated in V. vulnificus isolates in the MLST database. [file spectrum.00079-24-s0003.docx]

**Table S3** Alleles detected across all 10 genes evaluated in V. vulnificus isolates in the MLST database

| ***V. vulnificus*** | **Sources** | **Allelic profiles** | | | | | | | | | | **STs** |
| --- | --- | --- | --- | --- | --- | --- | --- | --- | --- | --- | --- | --- |
|  |  | ***glp*** | ***gypB*** | ***mdh*** | ***metG*** | ***purM*** | ***dtdS*** | ***lysA*** | ***pntA*** | ***pyrC*** | ***tnaA*** |  |
| C4 | Environmental isolate | 5 | **130** | **135** | **106** | 22 | **213** | **191** | 1 | **143** | **135** | 575 |
| C51 | Environmental isolate | **140** | **130** | **122** | **107** | **92** | **208** | **199** | 1 | **144** | **136** | 576 |
| CHAD1.2APW | Diseased fish isolate | 5 | **124** | **126** | **112** | 22 | **199** | **199** | 1 | **145** | **137** | 577 |
| CHAD1.7BD | Diseased fish isolate | **141** | **125** | **126** | **116** | 70 | **200** | **194** | 1 | **146** | **138** | 578 |
| CHAD2.11APW | Diseased fish isolate | **142** | **117** | **137** | **116** | 53 | **204** | **199** | 26 | **147** | **139** | 579 |
| CHOH2.11BD | Diseased fish isolate | **141** | **125** | **132** | **121** | 1 | **200** | **194** | **115** | **146** | **140** | 580 |
| DMST31751 | Clinical isolate | **146** | **117** | **129** | **109** | 23 | **215** | **187** | 36 | **149** | **137** | 581 |
| DMST31752 | Clinical isolate | **147** | **128** | **141** | **110** | 4 | **209** | **199** | 81 | **147** | **144** | 582 |
| DMST32989 | Clinical isolate | **148** | **117** | **123** | **123** | 4 | **210** | **186** | 26 | **147** | **145** | 583 |
| DMST3606 | Clinical isolate | **141** | **117** | **123** | **123** | 4 | **196** | **205** | 1 | **149** | **141** | 584 |
| DMST4183 | Clinical isolate | **145** | **129** | **134** | **112** | 20 | **203** | **189** | 87 | **150** | **142** | 585 |
| G16 | Environmental isolate | **149** | **130** | **136** | **107** | **92** | **211** | **190** | 1 | **154** | **146** | 586 |
| JASA1.6APW | Diseased fish isolate | **150** | **116** | **126** | **116** | 70 | **197** | **188** | 26 | **155** | **147** | 587 |
| K2 | Environmental isolate | **151** | **133** | **123** | **110** | 23 | **207** | **198** | 87 | **156** | **148** | 588 |
| VVA1 | Diseased fish isolate^a^ | **152** | **117** | **130** | **125** | 30 | **213** | **192** | 4 | **147** | **149** | 589 |
| VVA5 | Diseased fish isolate^a^ | 5 | **114** | **131** | **112** | 23 | **198** | **193** | 116 | **158** | **150** | 590 |
| VVA6 | Diseased fish isolate^a^ | **153** | **117** | **137** | **116** | 53 | **204** | **199** | 26 | **147** | **151** | 591 |
| VVA8 | Diseased fish isolate^a^ | **154** | **117** | **137** | **116** | 53 | **204** | **199** | 26 | **147** | **152** | 592 |
| VVA9 | Diseased fish isolate^a^ | **155** | **125** | **132** | **121** | 1 | **200** | **194** | **115** | **146** | **153** | 593 |
| W4.6 | Environmental isolate | **156** | **115** | **137** | **112** | 39 | **212** | **197** | **116** | **147** | **154** | 594 |

^a^ Fish pathogen, Bold numbers represent new allelic types.
